# Examining Perceptions Among Healthcare Providers on Their Awareness of and Experience with Prescribing and/or Referring Pre-Exposure Prophylaxis to Eligible Cisgender Black Female Patients: A Qualitative Inquiry

**DOI:** 10.3390/ijerph22030450

**Published:** 2025-03-18

**Authors:** Mandy J. Hill, Amber I. Sophus, Sarah Sapp, Jeffrey Campbell, Diane Santa Maria, Jamila K. Stockman

**Affiliations:** 1 Department of Population Health and Health Disparities, School of Public and Population Health, University of Texas Medical Branch at Galveston (UTMB), Galveston, TX 77555, USA; 2 Department of Health Promotion and Disease Prevention, Robert Stempel College of Public Health & Social Work, Florida International University, Miami, FL 33199, USA; asophus@fiu.edu; 3 School of Osteopathic Medicine, University of the Incarnate Word, San Antonio Campus, San Antonio, TX 78235, USA; sarahsapp96@gmail.com; 4 Allies in Hope Houston, Houston, TX 77030, USA; campbellj@aihhouston.org; 5 Department of Research, Cizik School of Nursing, University of Texas Health Science Center at Houston (UTHealth Houston), Houston, TX 77030, USA; diane.m.santamaria@uth.tmc.edu; 6 Department of Medicine, University of California, La Jolla, San Diego, CA 92093, USA; jstockman@health.ucsd.edu

**Keywords:** health communication, healthcare provider engagement, healthcare provider, PrEP access, cisgender Black women, PrEP uptake, HIV prevention, healthcare access, women’s health

## Abstract

Prescriptions for and use of pre-exposure prophylaxis (PrEP), an available and accessible HIV prevention strategy, remain low among cisgender Black women (CBW). Given PrEP is only available through a prescription from a licensed healthcare provider (HCP), there is a need to identify factors associated with HCP’s prescribing and/or referring PrEP to CBW. Qualitative methods (in-depth interviews) were used to examine factors shaping beliefs and behaviors among 12 HCPs that impact their willingness to prescribe or refer PrEP to CBW. Seven primary themes were identified during a thematic data analysis. The themes with the highest frequency of codes (fc) were the provider’s experience discussing sexual health (fc = 284), the provider approach to patient engagement (fc = 240), provider knowledge of PrEP (fc = 158), and the provider approach to determining PrEP eligibility (fc = 141). Findings indicate that prescribing and referral behaviors among HCPs can be influenced by their knowledge of PrEP; perceptions about PrEP for patients; comfort level in engaging/communicating with patients about PrEP; awareness of PrEP resources needed to improve PrEP access among patients; and patient–provider communication relative to sexual health, HIV vulnerability, and PrEP eligibility. Study findings illuminate how usual care practices contribute to gaps in PrEP access among CBW and highlight areas for intervention.

## 1. Introduction

HIV is a critical public health problem for cisgender Black women (CBW), who account for 50% of new HIV diagnoses among all women [1,2], despite representing 13% of the U.S. female population [2,3]. Regionally, this disparity is more pronounced in the South, where CBW have the highest HIV incidence rates among all females aged 13 and older [2]. This health inequity is driven by various social, structural, and institutional factors that are prominent in this region, including poorer general health, higher rates of poverty, and inconsistent availability and quality of healthcare services [4,5]. The introduction of pre-exposure prophylaxis (PrEP) as an available and accessible HIV prevention strategy has the potential to reduce HIV incidence rates among HIV-vulnerable populations, including CBW.

PrEP is an FDA-approved antiretroviral medication that reduces the risk of HIV acquisition among HIV-negative individuals [6,7,8,9,10,11,12,13,14]. PrEP is currently available in two modalities: an oral daily PrEP pill [15,16] and bi-monthly intramuscular long-acting injection [15,16,17]. When taken as prescribed, PrEP is over 95% effective. This level of effectiveness makes PrEP an important prevention tool in ending the HIV epidemic [16,18,19,20]. However, PrEP prescriptions and use among Black populations remain low, especially among women. Among all PrEP users in the U.S., 8% are women, despite women comprising 19% of all new HIV diagnoses [1]. CBW represent less than 2% of PrEP users [21]. Disparities in PrEP use are also dispersed regionally. Black Americans have the greatest unmet need for PrEP across all U.S. regions, specifically in the U.S. South, where Black Americans have the lowest PrEP-to-Need ratio (PnR: a measurement of whether PrEP use appropriately reflects the need for HIV prevention) compared to Americans of other races and ethnicities [1]. For instance, in 2023, there were 25 White PrEP users for every new HIV diagnosis among White Americans but only 5 Black PrEP users for every new HIV diagnosis among Black Americans and 8 Hispanic PrEP users for every new HIV diagnosis among Hispanic Americans [1]. To date, women still represent a lower proportion of PrEP users in the U.S. South than men [1]. These statistics underscore the importance of prioritizing CBW in HIV prevention and care efforts and enhancing the provision of PrEP for this population within healthcare settings.

CBW face unique barriers to PrEP use due to the intersection of multiple social locations (i.e., an individual’s position within society) and identities (i.e., a combination of factors like race, gender, class, sexual orientation, etc.) that work together to create hardship for them, including limited knowledge and awareness of PrEP [22,23,24,25,26,27], low perceived HIV risk, lack of health insurance [21,22,25,28,29,30,31,32,33,34,35,36,37,38], HIV-related stigma [39,40,41], PrEP-related stigma [42,43,44], syndemic conditions (e.g., racism, poverty, low education, behavioral health issues, partner violence, and homelessness) [45,46,47], and structural barriers related to lack of access to preventive sexual health services [48,49]. In addition, medical mistrust has emerged as a particularly significant and complex barrier to PrEP uptake among Black women [41,50,51,52,53,54]. For Black individuals, medical mistrust—the negative perceptions and attitudes individuals hold toward healthcare systems—stems from a history of unethical medical research practices (e.g., the Tuskegee Syphilis Study [55] and the story of Henrietta Lacks [56,57]), the current mistreatment of Black individuals in healthcare settings due to systemic racism (including both institutional and cultural forms) [58,59], harmful stereotypes [60,61], and experiences of discrimination [62]. For instance, Black women who perceive themselves as having experienced discrimination within healthcare settings may be uncomfortable discussing their want for PrEP or hesitate to disclose HIV risk behaviors that may establish the potential health benefit if they were to receive PrEP. These patient-specific barriers often intersect with and compound provider-related barriers, creating a complex landscape for PrEP implementation and prescribing practices among providers.

Given PrEP is only available through a prescription from a healthcare provider (HCP), HCPs (including prescribing nurses) serve as essential sources of PrEP information and access [23,36,44,63,64,65] and have the capacity to serve as change agents to improve PrEP uptake among CBW. Even though PrEP prescribing has increased over time, there is growing evidence that HCPs are not comfortable initiating or engaging in conversations about PrEP with CBW [39,40,66] and, thus, have exhibited low willingness to prescribe and/or refer PrEP. D’Angelo and colleagues [66] conducted a qualitative study about barriers to and facilitators of PrEP uptake among Black women. Women in their study revealed that their primary care physicians and obstetrician–gynecologists frequently neglected to provide them with information about PrEP, which could effectively safeguard them against HIV. Prior research indicates that HCPs are less willing to prescribe PrEP when they have limited PrEP knowledge [67,68], lack understanding of clinical PrEP guidelines [68], have implicit bias [67,69], and have racial bias based on assumptions about PrEP adherence expectations [70]. HCPs are also less likely to prescribe PrEP when they are uncomfortable taking sexual history [68,71,72], have concerns about PrEP-related costs [67,68,72], and/or have general concerns about poor PrEP adherence among patients [67,68,73]. Conversely, HCPs are more willing to prescribe PrEP when they are educated about PrEP [74,75,76,77,78], an expert in HIV (e.g., HIV clinician) [77,79,80,81], have had patients who are living with HIV [73,78,82], and have generally positive attitudes towards PrEP [77,78]. Despite these findings, willingness to prescribe PrEP and positive attitudes towards PrEP do not always translate to the prescribing of PrEP among HCPs [83,84].

Willingness to prescribe PrEP refers to a provider’s mindset rather than their actions, which may be shaped by personal beliefs, perceived patient need, knowledge, comfort with PrEP, or attitudes toward HIV prevention, whereas the behavior of actually writing a PrEP prescription can be affected by various barriers (e.g., institutional policies, patient access, insurance issues, and time constraints) that may prevent an HCP from doing so. For instance, Karris et al. [84] conducted a nationwide survey (N = 573) to assess provider opinions, readiness, and current practices related to PrEP in the U.S. and Canada. Findings revealed that although HCPs were willing to prescribe PrEP, only 9% had provided PrEP to their patients [84]. Reasons for not prescribing PrEP included worry about adherence and the risk for future resistance, worry about cost, and insufficient evidence for the efficacy of real-world PrEP use [84]. Similarly, Pratt et al. [83] conducted a study with HCPs (N = 36) caring for Black adolescent girls and young women. Findings indicated that while HCPs were willing to prescribe PrEP, only 11% had prescribed PrEP to a patient [83]. Reasons for not prescribing PrEP were low familiarity with the U.S. Centers for Disease Control and Prevention (CDC) guidelines and PrEP options and lack of provider training [83]. Overall, addressing factors that influence willingness alone may not lead to increased PrEP uptake. If HCPs are willing to prescribe or refer a patient for PrEP but do not, interventions must address multi-level factors, such as structural, logistical, and attitudinal barriers to address this gap in PrEP use. Altogether, these findings highlight the need to understand which factors influence PrEP prescribing and referral practices among HCPs in order to develop targeted interventions to improve PrEP uptake among CBW.

The study team recognizes the gap in literary evidence on the PrEP implementation intentions of HCPs who care for CBW. Addressing this gap is essential, as the active involvement of HCPs in prescribing PrEP is crucial for extending PrEP access to this population. As such, between July 2022 and August 2024, a developmental study was conducted to create and pilot a health communication intervention using video logs tailored to Black women and HCPs who treat and care for Black women to motivate actionable access to and uptake of PrEP [85]. In the current study, we report on the qualitative findings stemming from this developmental study, which aimed to qualitatively examine factors that shape HCPs’ beliefs and behaviors in prescribing or referring PrEP to CBW. The qualitative findings were used to develop health communication material from the perspective of HCPs to inform the content of the video log-based intervention [85].

## 2. Materials and Methods

This qualitative observational study was reviewed and approved by an institutional review board prior to participant enrollment (HSC-MS-21-0419). We recruited a purposive sample of prescribing HCPs who care for CBW as a part of their patient population through convenience sampling using electronic flyers (e-flyers) that were disseminated through social media channels of academic and community partners. In-person recruitment strategies were also used, including dissemination of the study flyer through the social networks of community-based agencies, clinics, and HCPs within partnering hospital systems of the study site’s academic center. All flyers contained a QR code that linked potentially eligible individuals to a screening tool housed in Qualtrics to assess their eligibility for study participation. To be eligible, an individual had to be credentialed with privileges to prescribe and/or refer patients to PrEP, ≥18 years of age, fluent in English, and have access to a phone or the Internet. HCPs who were unable or unwilling to meet study requirements were not eligible to participate. If eligible, individuals were prompted to provide their contact information (email address and phone number) and selected a date/time for the interview that aligned with the availability of the research coordinator (RC). The RC reached out to those who scheduled interviews prior to the interview date and time to explain the study aims, send the electronic consent from using DocuSign Release 2, 2024 (DocuSign, Inc., San Francisco, CA, USA), and send the weblink for the meeting where they could confirm their participation in an in-depth interview.

The semi-structured interview guide included open-ended questions about the influences to support changes in beliefs and behaviors related to increasing willingness with respect to the prescribing or referring PrEP to CBW. The Theory of Planned Behavior was used to justify these influences on changes in clinical care behaviors to include prescribing or referring PrEP to eligible patients. The Theory of Planned Behavior [86] includes the theoretical concept of perceived behavioral control and can be applied to prescribing or referring PrEP as a new prevention behavior. This theory not only accounts for factors that are evidenced to influence behaviors of HCPs (i.e., implementation intentions [87] to prescribe and/or refer PrEP) but also has flexibility for use with diverse populations of HCPs in varied clinical settings [88,89].

During the scheduled interview, the RC ensured participant names were replaced with a study identification number to maintain anonymity. The RC then began the interview, explained the study, and facilitated the discussion using the interview guide that was developed by the investigative team. All interview sessions were audio-recorded with transcription using videoconferencing software (i.e., Microsoft Teams, Microsoft Windows 11 v23H2, Redmond, WA, USA) or Zoom 5.15.2, Zoom Communications, Inc., San Jose, CA, USA) on a one-on-one basis to mitigate scheduling as a barrier to participation for HCPs with varied clinical schedules. Participants received a physical gift card valued at USD 50.00 via United States Postal Service mail. The RC completed 12 interviews with prescribing HCPs.

The study PI reviewed the recording of each interview within 7–10 days of the interview taking place. After the review of 10 interviews, the PI noticed that the emergence of new themes began to dwindle, indicating that the study was likely approaching the point of saturation. The study PI led a meeting with study team members regarding the lack of new emerging themes, and a consensus was reached to conclude study enrollment at 12 participants.

### Data Analysis

The screening tool garnered 123 responses. The first two screening questions confirmed that the respondent was an HCP and that they were credentialed with privileges to prescribe and/or refer PrEP for patients. We assessed demographic data at the individual level regarding age, biological sex, race, and ethnicity. We also inquired about fluency in English, phone/Internet access, geographic location, HCP type, clinical discipline, length of clinical career, and interest in interview participation. Most screener respondents were female, younger than 50 years of age, Black, African American, or Non-Hispanic/Latino; resided in Houston, TX; were a nurse practitioner or physician; practiced reproductive health or family medicine; and had 1–10 years of clinical practice experience (see Table 1). In comparison, most study participants were also female, younger than 50 years of age, Black, African American, or Non-Hispanic/Latino and resided in Houston, TX. The overwhelming majority of our participants were physicians in family medicine or emergency medicine with 1-10 years of clinical experience. Although the originally planned sample size for HCPs in this study was 20, data collection reached saturation with the enrollment of 12 participants.

The recordings of the interviews were professionally transcribed by a third-party transcription service. The RC cleaned the professionally transcribed transcripts, ensuring they were de-identified and that the transcribed text were labeled by the speaker (i.e., the interviewer or the participant). The three-member coding team co-created an initial codebook using one transcript during a series of virtual meetings using Microsoft Teams software. The team lead has robust experience analyzing qualitative data using thematic content analysis [90,91,92,93]. Once the preliminary codebook was developed, each coder (n = 3) blind-coded the transcripts separately. Then, coders came together when independent coding was complete, compared codes, discussed differences, and reached a consensus using a constant comparative analytic approach to revise the final codebook [94]. New codes were added only when new codes emerged. The updated codebook, with the addition of new codes, was reviewed by the team and duplicate codes were collapsed. Then, coded data was analyzed for emergent categories and themes [95], which guided content development for vlogs for HCPs in the next phase of this study. Themes were differentiated as dichotomous categories that likely inform the HCP’s decision to prescribe or refer PrEP to CBW, namely ‘self-awareness’ or ‘knowledge’. First, self-awareness is a foundational element of the self-concept theory [96]. The self-concept theory [96] describes an individual’s internal sense of who they are. It enables individuals to compare their actual selves with their ideal selves. In this context, the ideal self might be a clinician who provides patient-centered care, including preventive measures like prescribing or referring PrEP to prevent HIV acquisition among HIV-negative patients. Alternatively, it could be a clinician who focuses solely on addressing the patient’s immediate needs and treats the primary presenting condition. ‘Self-concept’ includes perception, awareness, comfort, concern, desire, and preference. These descriptive terms reflect a fundamental part of the human experience and how an individual understands and interprets sensory information. As such, ‘self-awareness’ allows HCPs to understand their own values and motivations, as these predisposing factors are key elements in their process of making a clinical decision. In this analysis, we assess and discuss self-awareness through the lens of HCPs as they relate to PrEP prescribing and referral behaviors.

Second, ‘knowledge’ is needed to inform clinical decisions to prescribe and refer PrEP. The elements of knowledge referred to in this category include experience, approach, training, and education. These descriptive terms reflect an essential part of the human experience whereby an individual applies knowledge to achieve practical goals and solve problems in the real world. However, ‘self-concept’ is missing from the reasoning process used to attain understanding, and the ‘self’ does not inform the interpretation of information. Altogether, themes are organized based on whether ‘self-awareness’ or ‘knowledge’ serves as the most influential predictor of whether or not HCPs will prescribe or refer PrEP to their patients who are CBW.

Using the IBM SPSS v 26.0 statistical software package, the team calculated a Kappa statistic to assess inter-rater agreement between coders and across all identified codes. The inter-rater reliability was assessed across 1217 total unique codes. The inter-rater agreement between coder 1 (665 codes used in the independent coding) and coder 2 (723 codes used in the independent coding session) was −0.227 and S.E = 0.004 across 1873 valid cases. The inter-rater agreement between coder 2 (723 codes used in the independent coding session) and coder 3 (485 codes used in the independent coding session) was −0.183 and S.E > = 0.004 across 1873 valid cases. The inter-rater agreement between coder 1 (665 codes used in the independent coding session) and coder 3 (485 codes used in the independent coding session) was −0.176 and S.E > = 0.004 across 1873 valid cases. This Kappa statistic infers that there was little to no difference between the observations of the coders and chance alone. The negative Kappa statistic across all three coders suggests no agreement. The team generated a report and conducted member checking with community partners during professional meetings held nationally, roundtable discussions, and presentations of preliminary data and concepts across the local sexual health community.

## 3. Results

The full thematic analysis of all interview transcripts garnered 1217 codes. All codes in this qualitative analysis were related to participating HCPs discussing their experiences with and perceptions about prescribing and/or referring PrEP to CBW patients. A total of 28 themes were initially identified in the thematic analysis and stratified based on whether the team perceived the code as stemming from ‘self-awareness’ or being related to ‘knowledge’ (see Appendix A, Table A1 and Table A2). Below, we focus on the top themes across each category (i.e., themes with the highest frequency of codes (fc) (see Table 2 and Table 3).

### 3.1. Self-Awareness

Themes for ‘self-awareness’ relative to HCP experiences with and perceptions about prescribing and/or referring PrEP to CBW were found to be provider awareness of PrEP facilitators (fc = 119), provider perceptions on PrEP (fc = 105), and provider comfort with engaging patients about PrEP (fc = 104) (see Table 2). All initial themes and associated codes related to ‘self-awareness’ are described in Appendix A, Table A1.

#### 3.1.1. Provider Awareness of PrEP Facilitators

Participants indicated having an awareness of the factors that encourage PrEP use, including prescriptions and how to expand PrEP access to HIV-vulnerable populations. For example, one participant described patient–provider communication as an integral factor in facilitating PrEP uptake.

*I think one of the first things that I think about is the level of communication. So, if as the patient, they don’t feel comfortable talking to their provider about it, then how can we ever know that they need it? And so, I think it goes, yes, it is important for the provider to provide a space for them to feel comfortable, but I also think the patient has to feel comfortable enough to discuss what’s going on and say, “Hey, this is my situation. This is what I’m going through”. And so, how do you provide that balance? How do you get them to feel comfortable, but then how do you get them to actually communicate? So, it’s just a two-way street.*

Participants also described different ways to expand access to PrEP among CBW, such as by increasing awareness and education about PrEP, addressing misinformation about PrEP, improving patient–provider communication, and improving PrEP messaging toward Black women. For instance, some participants stressed the need to improve PrEP education outreach efforts for CBW.

*I think it would have to be obviously community outreach, letting people know wherever they congregate–churches, shopping centers, schools, whatever–starting to increase the awareness of the medication.*

*It’s good knowledge for anybody. And I think that honestly, I know that Texas is very prudish in the way they have information that they give to the kids, especially during their repro health classes in school. But if they would include PrEP and what it’s being used for, I think it would have a better… These kids would have a better understanding.*

Some participants highlighted the need to expand the types of providers who know about and prescribe PrEP (i.e., OBGYNs) in order to expand access to PrEP among CBW.

*Bring it up in the pap smears or bring it up in the well women visits.*

*I would say reaching out to more of the OBGYNs.*

*Probably in the primary care or gynecological visits, offering it as just another form of protection against anything in the same way we offer birth control or the same way we encourage condom usage and things like that. Just another low side effects pill that can help prevent things.*

Participants also described their reasons for liking PrEP for patients, such as its high efficacy and effectiveness in preventing HIV among HIV-vulnerable communities, the different modalities offered for PrEP, and the affordability and availability of PrEP.

*…what I like about PrEP is it does decrease the risk of transmission, especially of at-risk populations. I like that we do have now two alternatives, a pill that’s for everyday use and a shot that’s once a week. I’ve seen the studies.*

*I believe it’s highly effective, from what I know, and the dosing, from also what I know, is much easier than when I was in medical school. It used to be very… Actually, there wasn’t even really such thing. We only had PrEP for people who got stuck with a needle, but for the general population who is at higher risk, from what I understand, the dosing is much easier than it used to be. So, it’s more accessible, I guess, is what I’m trying to say, and it’s been proven useful.*

*It’s simple. From what I know, it’s just one pill and it’s once a day. From what I know, like I said, there’s not too many side effects or too much further testing that has to be done afterwards. Yeah, I think those are the best things. It’s easily accessible or available. Most pharmacies carry it within a city and you don’t have to wait too long to get it versus some things have to be flown in and things like that.*

*Just the convenience of the patient being able to have access to it. And then also the funding available for patients. So, if they can’t afford it, then there’s funding available for them to be able to get the medication so that way they don’t have to go without.*

#### 3.1.2. Provider Perceptions on PrEP

Participants in this study had positive perceptions about PrEP. Participants described PrEP as an additional tool for women to use to improve their sexual health and sexual wellbeing such that PrEP is necessary and a great resource for preventing HIV among HIV-vulnerable populations.

*So, what I like about PrEP is that I feel like it empowers… In this case, it empowers my patients. It empowers women to be able to have something else in their toolkit or on their tool belt to help protect their sexual wellbeing, their overall wellness.*

*In terms of pre-exposure prophylaxis, I think it’s great for the community in terms of helping prevent the spread and helping those who are definitely in need. I think it’s going to be a great… Or it is a great opportunity for those who, especially, are maybe unsure that their partner has this, it can help them along the lines of that as well. Because I know sometimes people are afraid to discuss that specific disease state. And so, that is a great opportunity to help them with that as well, as a preventative measure.*

Participants also described how PrEP can be used in combination with other medication while providing protection to individuals who opt to use PrEP.

*I think it’s definitely a necessary medical treatment. When I have used it in the emergency department setting, usually it’s for someone that unfortunately has been sexually assaulted as part of a forensic nursing sort of thing. And I’ll ask patients if it’s something that they want. And usually I’m doing this in conjunction with a forensic nurse examiner and they will help me figure out the best sort of combination of drugs and everything and then we will either dose it in the ED or give them prescriptions to follow up with…*

Relative to the theme of ‘provider perceptions on PrEP’, rationale for why providers like PrEP for patients included factors related to the utility of PrEP. Participants described PrEP as being manageable and discreet and believed PrEP to be well-developed as an HIV prevention tool.

*I think the fact that it’s every three months is definitely manageable*

*…it doesn’t have to be anything that anybody knows about, that they can now have the injection, and it could be just during a regular doctor’s visit and no one would ever know.*

*I think those as a clinician, it’s what you want. You want something to be developed, you want something to be approved, and you want very clear indications of who needs it.*

#### 3.1.3. Provider Comfort with Engaging Patients About PrEP

Some participants were comfortable engaging with patients about PrEP, while others experienced discomfort identifying eligible patients. Participants described introducing PrEP during conversations about a patient’s sexual behaviors and activities, with providers’ level of ease in discussing PrEP dependent on their experience engaging with CBW and their perception of why PrEP may be good for a patient. For instance, some participants described using various clinical situations as opportunities to initiate PrEP conversations, which typically occurred when they suspected a patient to be vulnerable to HIV acquisition. As such, these conversations often occurred during discussions about HIV risk behaviors (i.e., having multiple sexual partners, frequent sexual activity, and HIV/STI testing).

*Probably when I go into testing and sexual history, so if they’re having frequent sex or frequent sexual partners or have suspicion that their partner is having sex with other sexual partners, I’ll bring up the different treatments and testing that we can do. And then probably when I mention getting an HIV and AIDS test, I’ll probably mention also if you’re concerned, you can get on PrEP, the once a day pill.*

*I would tell them like, “Hey, I noticed we’ve seen you a couple times in the clinic a lot of times for STD testing. Is it the same partner?” I will ask them is it the same partner? Is your partner stepping out on you or are you just having a lot of partners. She’s like, “Oh, a new boyfriend?” Or they say, “Oh, there’s new partners”. I’m like, okay, well, I’ll first bring up, “Are you using contraception? Are you using condoms?” That kind of stuff.*

*I actually just had one two days ago who came in… Brand new patient came in for vaccines, found out she was sexually active, recently moved to Texas. I can’t remember where she’s from. Turned out she had eight partners in the past. So, we discussed trying to limit partners. Obviously, using condoms to prevent pregnancy and STIs. Drew all the labs like I would for PrEP. Discussed PrEP with PrEP. She didn’t seem interested at the time. So, when she comes back to review all of her blood work, I will again address the conversation of trying to prevent HIV.*

Providers also described challenges in engaging CBW about PrEP, which may have limited their willingness to prescribe or refer PrEP to patients.

*I think I’ve only had one female patient that was Black that was interested in PrEP, and she was already taking it, and she was there for a refill. She was taking it because her partner was HIV positive. I won’t say I don’t know, but I don’t recall how the partner became positive. But I know it was a male partner, I remember that much. And she was telling me that, “Yeah, I’ve been on PrEP for a year or two, and I’m just here for the refill”. So, I haven’t had experience of some Black female patient coming in requesting being on PrEP. I have brought it up once or twice before, but they always turned me down because it was more along the lines of like it was basically patients that I suspected had history of multiple STDs, that kind of stuff.*

In the context of ‘provider comfort with engaging patients about PrEP’, participants described liking PrEP for patients given the role they play in prescribing it, such as decreasing pill burden, the potential to increase adherence, and ensuring patients are receiving the best care.

*I think it’s also something that I’m a fan of is that as pharmacists, we do play a role in this, by the pharmacology of the drug. And so, what we can do is we can help to decrease the pill burden, and we can help to decrease the amount of pills that patients take. Number one, increase adherence. We play a role in helping to assist with how can we ensure that our patients are getting the best care. And that while they are on these medications, ensuring that these medications are working properly, and that they’re not interacting with other medications that may be on their profile.*

#### 3.1.4. Provider Awareness of PrEP Resources

Participant’s awareness of PrEP resources varied. Although participants described various approaches to gain more knowledge about PrEP, participants lacked knowledge on specific resources needed by patients to access PrEP. For example, participants had limited knowledge and experience with payment assistance programs and, thus, lacked a comprehensive understanding of the cost landscape surrounding PrEP.

*I don’t know. I don’t have any knowledge on that, to be honest.*

*No clue about that, but I do have PrEPs for Descovy come in and they have coupon cards. So, I do have coupon cards for Descovy, kind of like a pay card.*

*I do know there are some payment assistance programs for patients with HIV out there, but I don’t know the specifics about it.*

Alternatively, participants reported various resources that could help them stay informed about PrEP, such as scholarly reviewed articles (i.e., PubMed), medical drug information databases (i.e., Lexicomp, Up To Date, etc.), websites that cater to providers (i.e., American Association of Family Physicians, Trusted Health Plan, etc.), and continuing medical education (CME) courses. These resources help providers enhance their knowledge of PrEP, stay updated on current information, and remain aware of any changes in PrEP guidelines or recommendations.

*So, on a day-to-day basis, we use databases like Lexicomp, Clinical Pharmacology, UpToDate, PubMed. All of these are databases that have information on medication. And so, as I mentioned throughout our interview, the field is ever evolving, especially with the drugs. And so, we use these databases to ensure, “Hey, I’m telling my patient the right thing. Hey, this is the first time I’m seeing this new combination of drugs, what I need to let them know about. These are the common side effects,” things along those lines.*

*All of the physicians have to do it. You have to continue to learn, so you have to show proof that you’ve continue to learn. We get lots of advertisements, posters, whatever, about different CME activities so you can sign up for one and learn about it.*

*I like to look at scholarly reviewed articles as opposed to just Googling stuff.*

Participants also mentioned conferences, departmental meetings, and lectures as additional resources to gain more knowledge about PrEP.

*So having the presentations, the pharm reps. I’m in the HIV organization. So, I learn information from there as well, and continuing medical education.*

*The reps come in, teach us a little here and there. They’ll bring us packets that kind of show us maybe how to get certain things approved.*

*I think really it would probably be through either continuing education or really department protocols or conferences like our departmental meetings is usually how we would, I think, more uniformly get it amongst a whole group.*

### 3.2. Knowledge

Themes with the highest frequency of codes for ‘knowledge’ relative to HCP experiences with and perceptions about prescribing and/or referring PrEP to CBW were provider experience discussing sexual health (fc = 284), the provider approach to patient engagement (fc = 240), provider knowledge of PrEP (fc = 158), and the provider approach to determining PrEP eligibility (fc = 141) (See Table 3). All initial themes and associated codes related to ‘knowledge’ are provided in Appendix A, Table A2.

#### 3.2.1. Provider Experience Discussing Sexual Health

Participants described using a variety of approaches when engaging patients in sexual behavior discussions, including encouraging open communication when patients have new sexual partners or emerging concerns, providing clear explanations, reinforcing information about medications, and normalizing sexual health discussions by integrating these conversations into routine care for all patients.

*I think it’s to, at the very beginning to not be judgmental and respectful, even if it’s not necessarily what you would do, but just everything that we do in medicine is that you’re not going to have the same experience as somebody else to just be like, non-judgmental, non-confrontational and I think be empathetic towards the other person. How would you want to be treated in that circumstance? I think the relationship part is really, really important here because if your patient is not comfortable with you, I don’t think that they will be forthcoming or receptive to your ideas.*

*Always tell them if they have a new sexual partner or if they have a concern, this is always a safe space. Just come back. I use that word a lot in people who are anxious, depressed, like, oh, this is a safe space. Feel free to come back. Let me know. Let me know if there’s any changes. And so those are, I think the way you introduce or the way you have that initial conversation about sex and how you respond will set the precedent for them being able to come back if they have any other concerns.*

Participants also recognized the importance of building rapport, especially in settings like emergency departments, where encounters may be brief. In this regard, participants described a variety of useful approaches to integrate sexual health discussions during patient visits, including focusing on patient education; encouraging patients to make informed decisions about their sexual health; incorporating brief but targeted questioning; and engaging in sexual health discussions at opportune times, such as when patients pick up prescriptions or have questions.

*I just think it’s important that everybody who comes in, regardless of age or gender or whatever, that they realize that their sexual health is important. And I tell them all the time, No one’s going to love you as much as you love yourself. So, if you have sex with somebody and they don’t have a condom, you have to say no. Because they’re just going to do it. So, I’ve tried very hard to get these kids to realize that sex is their choice. Who they have sex with and how they have sex with them is a 100% their choice. And don’t let anybody else make that decision for them.*

*For me, I always ask as we go, as I’m explaining, does that make sense? Do you have any questions? Is there anything that I can explain further for you? And then I’m a visual person, so I pull up visuals.*

In addition, providers emphasized the need to change standard practices around sexual health discussions, expressing the need to collect additional information about a patients’ sexual activities, in addition to knowing whether they are currently sexually active or not.

*Again, like I said, I think everybody needs to not just ask, Are you sexually active? Check a box and move on. I think everybody needs to stop and ask those questions. Who are you having sex? How are you having sex? And what are you doing for your own… What are you using? And I think that’s where a lot of people stop. I think they, Are you sexually active? Yep. Okay. And I don’t think they ever delve into what kind of sex and who they’re having sex with. And I think that needs to be standard practice.*

*I don’t do it specifically for one patient population over the other. I try to normalize talking about sex as part of the physical exam, for me, especially when patients come with issues that are related to that. I always ask my patients, “Are you sexually active, yes, no?” And then if they say yes, I always go like, “Okay, male, female partners? One partner? Multiple partners”. I always like to make sure I get a hold of that. Sometimes patients tell me, “Oh, yeah, one, multiple,” or “male, female, both”. Sometimes they don’t feel comfortable discussing it, and that’s okay.*

#### 3.2.2. Provider Approach to Patient Engagement

Participants described using various approaches for patient–provider communication about sexual health, HIV prevention, and PrEP. For example, some participants described using a non-judgmental approach, including positive reinforcement, providing a physical safe space for patients to have more in-depth conversations, and asking questions throughout the explanation process when discussing different sexual health topics.

*Always try to keep them open. I always ask my high-risk patients, I always try to ask them, like, “Hey, you feeling safe at home? Is anybody pressuring you to do stuff that you don’t feel comfortable doing?” I specifically do that with my younger patients, my 15 to 24, 25 patients. I guess it can happen to anybody at any age, but especially younger patients, I kind of tend to be a little bit more like, “Hey, no one’s pressuring you”. I know if a patient’s younger than 18 and are sexually active, I always like to ask them like, “Hey, how old is your partner? They’re not 30, right? They’re not in their 40s? All right. Cool”. Just making sure there’s nothing crazy going on.*

*For me, I always ask as we go, as I’m explaining, does that make sense? Do you have any questions? Is there anything that I can explain further for you? And then I’m a visual person, so I pull up visuals.*

#### 3.2.3. Provider Knowledge of PrEP

Participant knowledge about PrEP varied. Similar to participants’ lack of awareness of PrEP resources, participants were unaware of and/or unfamiliar with payment assistance programs for PrEP.

*I do know there are some payment assistance programs for patients with HIV out there, but I don’t know the specifics about it.*

*Yeah, that’s correct. I don’t have any experience with that.*

While participants described having knowledge about PrEP (in general), i.e., that it is for the prevention of HIV, participants lacked knowledge about the different types of PrEP currently offered (i.e., injectable PrEP).

*So, there’s not a whole lot of medicine that we can say, “Hey, this is going to stop this”. PrEP for HIV is one of those. And I think it’s super important that these patients realize that the risk is high, regardless of who you have sex with, whether it’s men, women, both.*

*that they can now have the injection, and it could be just during a regular doctor’s visit and no one would ever know. So, I just love the fact that it is available, that not only the oral, but now the injectable.*

*Today’s the first day I’ve heard about injectable PrEP, so I learned something new today.*

One participant, who had not prescribed PrEP, was not as familiar with PrEP as an HIV prevention strategy.

*I think it’s a good method of preventing HIV in populations that are higher risk. I personally haven’t prescribed it myself, so I’m not really familiar with it, but I’ve definitely heard about it. But I think it is a good thing for prevention.*

#### 3.2.4. Provider Approach to Determining PrEP Eligibility

Provider perceptions of HIV vulnerability (an eligibility criterion for PrEP) among patients may influence whether patients are determined to be a good candidate for PrEP and, thus, influence the prescribing behaviors of HCPs. For instance, approaches used to determine PrEP eligibility were primarily related to provider assessment of a patient’s sexual and/or medical history.

*I guess just based on the history. History and physical that we do. And then also the pre-exam questionnaire.*

*I always make sure to ask, “Hey, you’re here for your physical”. If it’s a well woman or a well male exam, “Oh, are you sexually active? Yes, no?” Like I said earlier, I ask a male, female, I’d like to make it not awkward to be like, “Oh, yeah, with who?” So male, female partners, one or multiple partners. Are you using protection, condoms, pills, whatever? That’s kind of where you really suss out the information of who’s going to be at risk, who’s going to be not at risk.*

Provider support for offering PrEP was specifically based on their perception of a patient’s risk. Participants in this study described routinely offering PrEP to adolescents and adults, primarily if the individual reported having multiple sexual partners, had a history of multiple STIs, and/or engaged in drug use.

*And I talk about PrEP probably once or twice a week with my high risk adolescents who have high number of partners, or men who have sex with men. I bring that conversation up every time I see them.*

*If they are at risk, so if they engage in risky sexual practices, I would consider them at risk.*

*I think anybody who’s at risk. So multiple sexual partners or anyone or patients who are just… No curious but requested and after proper discussion with them, if they are a good candidate, I think they should be able to get it.*

*…every July one, we re-administer that risk assessment. However, I typically ask all of my patients anytime they follow up, I go through our same… Ask if there’s any change in their medical history, allergies, family history. And then I always readdress any smoking, drugs or alcohol. And are you sexually active? So, even though we don’t do a formal evaluation of risk, except once a year, I check risk pretty much at every visit. Those particular, the big ones. Smoking, drugs, alcohol and sexual activity.*

Relatedly, participants discussed their strategies used for assessing risk and expressed the need to improve risk assessment methods, noting that simply asking whether a patient is sexually active is insufficient. Some participants emphasized the importance of asking additional questions to fully understand a patient’s vulnerability to HIV, such as including detailed questions about sexual partners, practices, and protection methods, in standard practice.

*… I think everybody needs to not just ask, Are you sexually active? Check a box and move on. I think everybody needs to stop and ask those questions. Who are you having sex? How are you having sex? And what are you doing for your own… What are you using? And I think that’s where a lot of people stop. I think they, Are you sexually active? Yep. Okay. And I don’t think they ever delve into what kind of sex and who they’re having sex with. And I think that needs to be standard practice.*

*I talk about risk all day long. We do what we call a wraps, which is like a 21 question risk assessment that goes all the way from, do you eat fruits and vegetables every day? To self-harm, sexual activity and everything. So, any child that says, yes, that they are sexually active, I discuss type of partners, male, female, both. I discuss what parts of your body do you use to have sex? So, that way, I can gauge what their risk is.*

*Again, I just start out the conversation with… And every time they come, my first questions are after I’ve seen them, have you had any unprotected sex since your last visit? Have you had any new partners since your last visit? And if they tell me no, then I ask them [if they’ve] had any sexual activity at all since your last visit. And it doesn’t matter if they’re here for a hepatitis vaccine, or if they’re here because they have an earache. Any of my sexually active patients, I discuss sexual activity at every visit. So that way, like I said, if I stayed enough times then maybe they’ll believe me that it’s important.*

## 4. Discussion

The purpose of this qualitative study was to explore factors that influence beliefs and behaviors related to the decision to prescribe or refer PrEP to CBW among a small cohort of HCPs. As the incidence of HIV remains a significant public health concern, particularly among HIV-vulnerable populations, HCPs must be equipped to readily extend PrEP access and to prescribe it effectively. Findings from this qualitative study indicate that prescribing and referral behaviors among HCPs can be improved by increasing provider knowledge about PrEP; perceptions about PrEP for patients; comfort level with respect to engaging/communicating with patients about PrEP; awareness of PrEP resources needed to improve PrEP access among patients; and patient–provider communication relative to sexual health, HIV vulnerability, and PrEP eligibility. Normalizing PrEP discussions led by HCPs during routine care, increasing provider education on the different PrEP modalities and PrEP resources available to patients, improving risk assessment protocols, and expanding referral networks could be pivotal in decreasing the PrEP use gap among CBW.

Specific knowledge about PrEP, including PrEP medication and PrEP resources, has been highlighted in prior work as a critical factor in HCPs’ willingness to prescribe PrEP [68,97,98]. In the current study, HCPs lacked knowledge about payment assistant programs, a primary resource needed for patients to access PrEP. Depending on a patient’s financial status, medication costs can vary significantly. High out-of-pocket costs may force individuals who are good candidates for PrEP and who are interested in PrEP to have a decreased interest in this highly effective preventive tool. Knowledge of payment assistant programs, including generic options, can help HCPs support patient access to PrEP and allows them to better advocate for patients who experience financial barriers to health care [99]. By being aware of and understanding the financial aspects and resources associated with PrEP use, HCPs are in a better position to help patients navigate the complexities associated with accessing PrEP.

Key themes in this study are reflective of patient–provider communication, primarily as they relate to HCPs’ ability to determine a patient’s vulnerability to HIV acquisition and their comfort discussing sexual health and engaging patients about PrEP. Prior research has also indicated low comfort levels and limited competency among providers in terms of discussing HIV risk and PrEP with Black women [100,101]. In the current study, HCPs primarily reported engaging patients about PrEP during discussions of sexual risk behaviors, often emphasizing a patient’s risk in order to influence PrEP uptake. This finding may indicate a need to change the way HCPs make decisions about who to engage and how to engage CBW in discussions about PrEP within healthcare settings. The PrEP guidelines [16] state that all “sexually active adults and adolescents should be informed about PrEP for prevention of HIV acquisition. This information will enable patients to both respond openly to risk assessment questions and to discuss PrEP with persons in their social networks and family members who might benefit from its use”. However, framing PrEP as solely for those engaging in “high-risk” sexual behaviors may deter Black women. Furthermore, the ideology that comes from this frame may inform language that may further alienate CBW who have experienced stigma at the intersection of medical mistrust [41,49,52,59,61] and the knowledge of historical medical injustices among Black populations. This approach can suggest that PrEP is only for individuals who engage in high-risk sexual behaviors, thereby stigmatizing PrEP use and excluding those who seek protection for other reasons (i.e., having an HIV-positive or abusive partner or to reduce worry of potential exposure to HIV in order to increase sexual pleasure). HCPs in this study recognized the need to ask additional questions to fully understand a patient’s “risk” beyond whether an individual is sexually active. Highlighting how PrEP could improve intimacy and desire could help PrEP appear more enticing to CBW who are prioritizing their sexual health. This is particularly important given the history of Black women being oversexualized [102,103,104], limiting their expression of sexuality and sexual desire. Moreover, focusing solely on sexual risk behaviors excludes other priority populations for HIV prevention (i.e., substance misusers) and fails to include the range of prevention options available to CBW (e.g., PrEP, PEP, syringe exchange, and condoms). Given that the updated PrEP guidelines [16] insist that PrEP should be prescribed to any individual who asks for it, regardless of their individual risk, updates are needed in the way CBW are engaged about their HIV “risk” and PrEP eligibility.

Study findings demonstrate a need for behavioral interventions that can motivate HCPs to lead sexual health discussions with CBW to bridge the gap between PrEP eligibility, access, and initiation among this HIV-vulnerable group of women. In this regard, thematic findings were used to inform health messages that will serve as the basis for new video logs that will be tested with HCPs through a randomized controlled trial as part of an intervention. The goal of this intervention is to enhance access to PrEP by increasing HCPs’ comfort with and willingness to prescribe and refer PrEP to CBW, culminating in the final phase to pilot the intervention and assess whether the intervention facilitates behavior change (i.e., if there is an increase in prescriptions written and referrals given to cisgender Black female patients over a 30-day follow-up period).

### 4.1. Implications for Healthcare Practice

The study findings have several implications for healthcare practice and policy. First, improving HCP knowledge about the different PrEP modalities (oral and injectable PrEP) and available resources for patients to access PrEP will help improve their comfort discussing PrEP with patients and, in turn, improve their PrEP prescribing behaviors among vulnerable populations like CBW. Providing training opportunities on effective communication strategies and providing comprehensive PrEP educational tools could be beneficial for HCPs. Participants in this study described using a variety of resources to gain more knowledge about PrEP (i.e., scientific articles or continuing medical education courses). This information can be leveraged for intervention development to improve PrEP prescribing behaviors among HCPs. Findings also indicate the need to improve and implement protocols assessing HIV vulnerability that extend beyond asking if a patient is sexually active. This could be particularly useful if there is an expansion of the type of HCPs who can prescribe PrEP. In the current study, participants suggested having PrEP offered within various clinical settings beyond primary care. Public health collaborations between health departments, emergency departments, community-based organizations, reproductive health clinics, HCPs (i.e., OBGYNs), and clinics can support a comprehensive and accessible PrEP care system. Broader PrEP referral networks can work to expand reach to priority populations, increase access points to obtain PrEP, and ensure patients who may not engage with healthcare systems regularly have access to this HIV prevention tool.

### 4.2. Limitations

For the current study, due to data saturation, we ended recruitment and data collection after enrolling 12 participants. The small sample size and geographic location in which this study took place limit the generalizability of the findings. Despite these limitations, the study findings highlight a broad understanding of HCPs’ experiences with and perceptions about prescribing and/or referring PrEP to CBW in diverse settings. Due to self-selection, the majority of HCPs in this study were physicians, limiting our understanding of factors that influence PrEP prescribing and referral behaviors among other types of providers. Future research should aim to purposely recruit different types of HCPs across different disciplines to identify potential variations in PrEP prescribing and referral behaviors among different types of HCPs (i.e., physicians, nurses, and pharmacists). It is also important to note that the current study did not aim to—nor did it—explicitly collected data about factors influencing PrEP prescribing and referral behaviors among HCPs who actively prescribe PrEP compared to those who do not. Future research that aims to make comparisons between groups should recruit HCPs who have and have not prescribed PrEP to CBW. Comparing the findings from these groups would provide valuable insights about their PrEP knowledge, attitudes, motivations, and access to PrEP-related resources. It would also help determine how specific factors may vary across different healthcare settings, enabling a broader understanding of how to improve PrEP access and utilization for CBW in diverse clinical environments. Despite conducting multiple coding sessions, both online and face-to-face, the research team’s final evaluation using the Kappa statistic did not show sufficient agreement between coders. This lack of consensus among coders potentially limits the broader applicability of the findings.

## 5. Conclusions

The current study explored factors that influence beliefs and behaviors related to willingness to prescribe or refer PrEP to CBW among HCPs who care for CBW as a part of their patient population. Findings emphasize the need for comprehensive provider education on PrEP, improved risk assessment protocols, and strategies to normalize sexual health and PrEP discussions in routine care. Enhancing patient–provider communication about sexual health and HIV vulnerability is a critical step in bridging the gaps between PrEP eligibility, access, and initiation among CBW.

## Figures and Tables

**Table 1:** Sample characteristics recorded from the individuals who completed the eligibility screener compared to study participants.

Variables	Subcategories	Respondents (N = 123)	Respondents %	Study Participants (N = 12)	Study Participants %
**Biological Sex**		N = 69	%	N = 12	%
	Female	45	65.22%	9	75.00%
	Male	20	28.99%	3	25.00%
	Non-binary/ third gender	3	4.35%	0	0.00%
	Prefer not to say	1	1.45%	0	0.00%
**Age**		N = 54		N = 12	
	<50 years	57	89.06%	11	91.70%
	>50 years	7	10.94%	1	8.30%
**Race**		N = 69		N = 12	
	White	6	8.70%	4	33.33%
	Black or African American	57	82.61%	5	41.70%
	American Indian or Alaska Native	2	2.90%	0	0.00%
	Asian	1	1.45%	1	8.30%
	Other	3	4.35%	2	16.77%
**Ethnicity**		N = 64		N = 12	
	Hispanic/Latino	15	23.44%	2	16.77%
	Non-Hispanic/Latino	49	76.56%	10	83.33%
**City of Residence**		N = 58		N = 10	
	Houston, TX	58	95.09%	10	83.33%
	Missing	3	4.92%	2	16.77%
**English Speaking**		N = 70		N = 12	
	Yes	69	98.57%	12	100.00%
	No	1	1.43%	0	0.00%
**Healthcare Provider**		N = 107			
	Yes	77	71.93%	12	100.00%
	No	30	28.04%	0	0.00%
**Type of Healthcare Provider**		N = 64		N = 12	
	Nurse Practitioner	19	29.69%	2	16.77%
	Advanced Practice Provider (APP)	1	1.56%	0	0.00%
	Physician	19	29.69%	8	66.77%
	Nurse	17	26.56%	1	8.33%
	Patient-Facing Pharmacist	8	12.50%	1	8.33%
**Healthcare Discipline**		N = 64		N = 12	
	Reproductive Health	25	39.06%	1	8.33%
	Family Medicine	18	28.13%	5	41.77%
	Internal Medicine	4	6.25%	0	0.00%
	Emergency Medicine	7	10.94%	4	33.33%
	FQHCs, Local Clinics, or Urgent Care Clinics	7	10.94%	0	0.00%
	Other	3	4.69%	2	16.77%
**Length of Career as a Provider**		N = 64		N = 12	
	<1 year	6	9.38%	2	16.77%
	1–5 years	22	34.38%	4	33.33%
	6-10 years	26	40.63%	4	33.33%
	11-20 years	6	9.38%	1	8.33%
	>20 years	4	6.25%	1	8.33%
**Access to a phone and/or Internet**		N = 69		N = 12	
	Yes	69	100.00%	12	100.00%
	No	0	0.00%	0	0.00%
**Credentialed to prescribe/refer PrEP**		N = 76		N = 12	
	Yes	69	90.79%	12	100.00%
	No	7	9.21%	0	0.00%
**Willing to participate**		N = 63		N = 12	
	Yes	62	98.41%	12	100.00%
	No	1	1.59%	0	0.00%

**Table 2:** Themes and associated codes identified through thematic analysis of interview data specific to ‘self-awareness’.

#	*Self-Awareness*	Theme	Codes	Frequency of Codes
1	**Awareness**	Provider awareness of PrEP facilitators		
			Provider perceptions on how to expand access to PrEP in cisgender Black women	29
			Provider’s perceptions on why they like PrEP for patients	25
2	**Perception**	Provider perceptions on PrEP		
			Provider perceptions on PrEP	45
			Provider’s perceptions on why they like PrEP for patients	25
3	**Comfort**	Provider comfort with engaging patients about PrEP		
			Provider experience in engaging Black female patients with PrEP	38
			Provider’s perceptions on why they like PrEP for patients	25

**Table 3:** Themes and associated codes identified through thematic analysis of interview data specific to ‘knowledge’.

#	*Knowledge*	Theme	Codes	Frequency of Codes
1	**Experience**	Provider’s experience discussing sexual health		
			Provider approaches to sexual health discussions	107
			Provider approach in engaging patients in sexual behavior discussions	85
2	**Approach**	Provider approach to patient engagement		
			Provider approaches to sexual health discussions	192
			Provider’s self-perception of approach to clinical care	11
3	**Knowledge**	Provider knowledge of PrEP		
			Provider lack of knowledge on payment assistance programs	22
			Provider knowledge of PrEP as HIV prevention	17
			Provider lack of knowledge on PrEP injectable	16
4	**Approach**	Provider approach to determining PrEP eligibility		
			Provider’s strategy for assessing risk	38
			Provider support for offering PrEP is based on their perception of patient risk	21

## Data Availability

The data presented in this study are not available because the data are qualitative and the words, the situations presented, and phrasing of the wording can potentially identify participants and compromise confidentiality.

## Appendix A

**Table A1:** All themes and associated codes identified through thematic analysis of focus group data specific to ‘self-awareness’.

#	*Self-Awareness*	Theme	Codes (n = 1217)	Frequency of Codes
1	**Awareness**	Provider awareness of PrEP facilitators		**119**
			Provider perceptions on how to expand access to PrEP among cisgender Black women	29
			Provider perceptions on why they like PrEP for patients	25
			Provider support for offering PrEP is based on their perception of patient risk	21
			Provider perception on what makes it easier to prescribe PrEP to patients	10
			Provider perception on facilitators of PrEP use in patients	7
			Provider perceptions on expanding PrEP access in healthcare systems	7
			Provider perceptions around patient preferences for injections versus pills	6
			Provider self-awareness of bias towards patients	6
			Reason why provider would feel motivated to prescribe PrEP	6
			Provider reliance on academic resources for PrEP knowledge	2
2	**Perception**	Provider perceptions on PrEP		**105**
			Provider perceptions on PrEP	45
			Provider perceptions on why they like PrEP for patients	25
			Provider perceptions on how patients would perceive PrEP side effects	16
			Provider perspective on PrEP within clinical practice	6
			Reason why provider would feel motivated to prescribe PrEP	6
			Provider’s perceived knowledge of PrEP side effects	4
			Provider perceptions that cisgender women are less likely to be referred to PrEP	3
3	**Comfort**	Provider comfort with engaging patients about PrEP		**104**
			Provider experience in engaging Black female patients with PrEP	38
			Provider perceptions on why they like PrEP for patients	25
			Provider leading PrEP discussions with patients	6
			Provider self-awareness of bias towards patients	6
			Provider self-motivation to prescribe more PrEP	6
			Provider persistence in leading preventative discussions with patients	4
			Provider willingness to discuss PrEP	4
			Provider educating patients on PrEP eligibility	3
			Provider lack of experience engaging Black female patients for PrEP	3
			Provider comfort in prescribing PrEP	3
			Provider comfort with engaging patients about PrEP	2
			Provider discomfort in identifying PrEP-eligible patients	2
			Provider willingness to engage in PrEP discussion to lower stigma	2
4	**Awareness**	Provider awareness of PrEP resources		**80**
			Provider use of resources to gain more knowledge on PrEP	28
			Provider lack of knowledge on payment assistance programs	22
			Provider lack of experience with payment assistance programs	13
			Provider knowledge of local clinics that offer PrEP	4
			Provider referral of patients to PrEP clinics with lower costs	4
			Provider use of marketing materials for PrEP	4
			Provider lack of knowledge on health insurance and PrEP	4
			Provider knowledge of resources to help patients get access to PrEP	1
5	**Desire**	Provider desire for direction and more knowledge on PrEP delivery		**76**
			Provider perceptions on how to expand access to PrEP among cisgender Black women	29
			Provider use of resources to gain more knowledge on PrEP	28
			Provider perceptions on expanding PrEP access in healthcare systems	7
			Provider self-motivation to learn more about PrEP	4
			Provider willingness to stay informed on PrEP	3
			Provider desire to learn more about prescribing PrEP	2
			Provider desire for PrEP guidelines within clinical care	2
			Provider desire for guidelines and resources on PrEP injectables	1
6	**Awareness**	Provider awareness of PrEP-related costs		**64**
			Provider knowledge of payment assistance programs	15
			Provider awareness of cost as a barrier to PrEP access	11
			Provider experience with insurance for PrEP	6
			Provider perception of health insurance and PrEP	6
			Provider experience with PrEP payments	5
			Provider limited knowledge on PrEP insurance coverage	5
			Provider experience with health insurance covering PrEP	5
			Provider referral of patients to PrEP clinics with lower costs	4
			Provider lack of knowledge on health insurance and PrEP	4
			Provider self-awareness of gap in knowledge of PrEP-related costs	2
			Provider experience with payment assistance programs	1
7	**Perception**	Provider’s perception of clinical peers engaging patients in sexual health		**64**
			Provider perceptions on clinical peers’ knowledge of PrEP	27
			Provider perception on standard practices of healthcare providers for sexual health	13
			Provider perceptions of gaps in delivery of preventive care amongst clinical peers	13
			Provider perception of low PrEP knowledge amongst clinical peers	6
			Knowledge of clinical peers offering PrEP services	4
			Provider perception of low PrEP knowledge among self and clinical peers	1
8	**Awareness**	Provider awareness of PrEP barriers		**62**
			Provider perception on barriers for PrEP use in patients	17
			Provider perspective on barriers to prescribing PrEP	14
			Provider perceptions on what may limit willingness to prescribe PrEP	12
			Provider reasoning for dislike of PrEP for patients	4
			Provider perceptions that cisgenders are less likely to be referred to PrEP	3
			Provider self-awareness of gaps in their own preventive care delivery	3
			Provider perception that men who have sex with men (MSM) are more comfortable referring PrEP	2
			Provider perception on why access to care varies in other populations	2
			Provider lack of a PrEP-eligible population due to specialty	2
			Provider uncertainty of forms of PrEP for cisgender women	2
			Provider uncertainty of when to offer PrEP	1
9	**Awareness**	Provider awareness of sexual risk behaviors within patient population		**57**
			Provider strategy for assessing risk	38
			Support of PrEP use is based on perception of risk	9
			Provider discussing PrEP with high-risk populations	3
			Provider awareness of high-risk behaviors within served population	2
			Provider support of STD treatment based on perception of risk	2
			Provider awareness of high-risk behaviors within served population	2
			Provider awareness of serving high-risk patient population	1
10	**Awareness**	Provider awareness of unconscious bias		**50**
			Provider experience with unconscious bias trainings	20
			Provider self-awareness after unconscious bias training	14
			Provider self-perception after unconscious bias training	6
			Provider perceptions that cisgenders are less likely to be referred PrEP	3
			Provider perception that MSM are more comfortable referring PrEP	2
			Provider willingness to engage in PrEP discussion to lower stigma	2
			Provider perception on why access to care varies in other populations	2
			Provider self-awareness of potential biases	1
11	**Awareness**	Provider awareness of stigma associated with PrEP		**38**
			Provider support of offering PrEP is based on their perception of patient risk	21
			Provider awareness of stigma associated with PrEP	8
			Provider awareness of stigma behind PrEP	5
			Provider willingness to engage in PrEP discussion to lower stigma	2
			Provider perception on why access to care varies in other populations	2
12	**Awareness**	Provider awareness of requirements of preventive care		**35**
			Provider broadens preventive considerations when clinical symptoms are absent and sexual risk is known	17
			Provider awareness on importance of patient follow-ups for preventative measures	6
			Provider awareness that follow-up care may be needed with PrEP	5
			Provider experience with patient referrals after prescribing PrEP	5
			Provider approach to follow-ups	2
13	**Awareness**	Provider awareness of social determinants of health		**24**
			Provider knowledge of payment assistance programs	15
			Provider experience with insurance for PrEP	6
			Provider awareness of socioeconomic status (SES) as a social determinant of health (SDoH)	2
			Provider experience with payment assistance programs	1
14	**Perception**	Provider perception of increasing health literacy in patients		**16**
			Provider perception on increasing health literacy in patients	16
15	**Awareness**	Provider awareness of patient comfort level		**15**
			Provider perceptions around patient preferences for injections versus pills	6
			Provider perceptions of patient population’s interest in PrEP	4
			Provider awareness that patient comfort levels vary based on provider gender	2
			Provider providing a comfortable space for patients	2
			Provider expecting patient to lead sexual health discussion	1
16	**Perception**	Provider perception of standard sexual health practices		**14**
			Provider perception on standard sexual health practices	14
17	**Preference**	Provider preference for patients to lead sexual health discussions		**6**
			Provider preference for patient to self-identify need of PrEP	2
			Support of PrEP is based on patient concern	2
			Provider preference for patients self-identifying as PrEP-eligible	1
			Provider expectation for patient to lead sexual health discussion	1
18	**Perception**	Provider perspective on dispensing PrEP		4
			Pharmacist perspective on process of dispensing PrEP to patients	4
19	**Concern**	Provider concerns about PrEP side effects		**2**
			Provider concern for renal disease and kidney function due to PrEP in Black community	2

**Table A2:** All themes and associated codes identified through thematic analysis of focus group data specific to ‘Knowledge’.

#	*Knowledge*	Theme	Codes (n = 1217)	Frequency of Codes
1	**Experience**	Provider experience discussing sexual health		**284**
			Provider approaches to sexual health discussions	107
			Provider approach in engaging patients in sexual behavior discussions	85
			Provider experience in engaging Black female patients with PrEP	38
			Experience discussing sexual health with Black female patients	16
			Provider leading PrEP discussions with patients	6
			Provider approach to HIV prevention discussions	4
			Provider educating patients on PrEP eligibility	4
			Provider discussing PrEP with high-risk populations	3
			Provider lacks experience engaging Black female patients for PrEP	3
			Provider leading sexual health discussion is based on clinical symptoms being present	3
			Provider comfort with engaging patients about PrEP	2
			Provider discomfort in identifying PrEP-eligible patients	2
			Provider experience discussing PrEP	2
			Provider lack of experience discussing sexual health with Black female patients	2
			Provider not discussing PrEP with patients	2
			Provider willingness to engage in PrEP discussion to lower stigma	2
			Experience discussing sexual health with African American population	1
			Provider expectation for patient to lead sexual health discussion	1
			Provider experience discussing sexual health with lesbian patients	1
2	**Approach**	Provider approach to patient engagement		**240**
			Provider approaches to sexual health discussions	192
			Provider self-perception of approach to clinical care	11
			Provider approach to engaging patients in discussion	8
			Provider leading PrEP discussions with patients	6
			Provider approach to HIV prevention discussions	4
			Provider persistence in leading preventative discussions with patients	4
			Provider leading sexual health discussion is based on clinical symptoms being present	3
			Provider self-awareness of gaps in their own preventive care delivery	3
			Provider providing a comfortable space for patients	2
			Provider support of STD treatment based on perception of risk	2
			Provider willingness to engage in PrEP discussion to lower stigma	2
			Provider approach to follow-ups	2
			Provider self-awareness of potential biases	1
3	**Knowledge**	Provider knowledge of PrEP		**158**
			Provider lack of knowledge on payment assistance programs	22
			Provider knowledge of PrEP as HIV prevention	17
			Provider lack of knowledge on PrEP injectable	16
			Provider knowledge on PrEP oral pills	15
			Provider knowledge of PrEP delivery routes	14
			Provider knowledge of PrEP side effects	12
			Provider knowledge on PrEP-related costs	12
			Provider perspective on who is eligible for PrEP	10
			Provider uncertain of PrEP knowledge	6
			Provider perception of health insurance and PrEP	6
			Provider lack of knowledge of PrEP side effects	5
			Provider reasoning for dislike for PrEP for patients	4
			Provider lack of knowledge on health insurance and PrEP	4
			Provider knowledge of PrEP	2
			Provider reliance on academic resources for PrEP knowledge	2
			Provider knowledge on PrEP oral pills	2
			Provider knowledge of side effects of oral PrEP	2
			Provider uncertainty of forms of PrEP for cisgender women	2
			Provider knowledge of resources to help patients get access to PrEP	1
			Provider limited knowledge of PrEP delivery routes	1
			Provider uncertainty of when to offer PrEP	1
			Provider limited knowledge on PrEP side effects	1
			Provider perceived knowledge of PrEP side effects	1
4	**Approach**	Provider approach to determining PrEP eligibility		**141**
			Provider strategy for assessing risk	38
			Provider support of offering PrEP is based on their perception of patient risk	21
			Provider perception on who is PrEP-eligible	17
			Willingness to offer PrEP to cisgender patients	16
			Provider approach to determining PrEP eligibility	13
			Provider self-perception of approach to clinical care	11
			Support of PrEP use is based on perception of risk	9
			Provider priorities during clinical discussions	8
			Provider priorities during clinical visit	6
			Willingness to prescribe PrEP to Black women	2
5	**Experience**	Provider experience prescribing PrEP		**56**
			Provider experience prescribing PrEP	20
			Provider lack of experience prescribing PrEP	15
			Provider perception on what makes it easier to prescribe PrEP to patients	10
			Provider self-motivation to prescribe more PrEP	6
			Provider comfort in prescribing PrEP	3
			Provider prescribing PrEP is based on availability	2
6	**Knowledge**	Provider knowledge gaps with respect to PrEP		**15**
			Lack of provider knowledge of PrEP-related costs	8
			Knowledge gap between PrEP and PEP indications	4
			Provider self-awareness of gap in knowledge with respect to PrEP-related costs	2
			Provider limited knowledge of PrEP delivery routes	1
7	**Training**	Provider HIV training focused on treatment		**3**
			Provider experience with counseling on HIV medication	2
			Provider training focused more on HIV medication counseling	1
8	**Experience**	Provider experience with patients taking PrEP		2
			Provider experience with patients using PrEP for prevention	2
9	**Education**	Provider education through research participation		**1**
			Provider learning new information from research interview	1
